# The number of measurements needed to obtain high reliability for traits related to enzymatic activities and photosynthetic compounds in soybean plants infected with *Phakopsora pachyrhizi*

**DOI:** 10.1371/journal.pone.0192189

**Published:** 2018-02-13

**Authors:** Tássia Boeno de Oliveira, Leonardo de Azevedo Peixoto, Paulo Eduardo Teodoro, Amauri Alves de Alvarenga, Leonardo Lopes Bhering, Clara Beatriz Hoffmann Campo

**Affiliations:** 1 Universidade Federal de Viçosa, Viçosa, MG, Brasil; 2 Universidade Federal de Lavras, Setor de Fisiologia Vegetal, Lavras, MG, Brasil; 3 Empresa Brasileira de Pesquisa Agropecuária (EMBRAPA), National Soybean Research Center. Rodovia Carlos João Strass, Access Orlando Amaral Warta, Londrina, PR, Brazil; Karnatak University, INDIA

## Abstract

Asian rust affects the physiology of soybean plants and causes losses in yield. Repeatability coefficients may help breeders to know how many measurements are needed to obtain a suitable reliability for a target trait. Therefore, the objectives of this study were to determine the repeatability coefficients of 14 traits in soybean plants inoculated with *Phakopsora pachyrhizi* and to establish the minimum number of measurements needed to predict the breeding value with high accuracy. Experiments were performed in a 3x2 factorial arrangement with three treatments and two inoculations in a random block design. Repeatability coefficients, coefficients of determination and number of measurements needed to obtain a certain reliability were estimated using ANOVA, principal component analysis based on the covariance matrix and the correlation matrix, structural analysis and mixed model. It was observed that the principal component analysis based on the covariance matrix out-performed other methods for almost all traits. Significant differences were observed for all traits except internal CO_2_ concentration for the treatment effects. For the measurement effects, all traits were significantly different. In addition, significant differences were found for all Treatment x Measurement interaction traits except coumestrol, chitinase and chlorophyll content. Six measurements were suitable to obtain a coefficient of determination higher than 0.7 for all traits based on principal component analysis. The information obtained from this research will help breeders and physiologists determine exactly how many measurements are needed to evaluate each trait in soybean plants infected by *P*. *pachyrhizi* with a desirable reliability.

## Introduction

Soybean (*Glycine Max* (L.) Merrill) is one of the most important cereal crops worldwide. Originally from East Asia, it is currently cultivated around the world for a variety of uses in animal and human food and for industrial applications such as oil and biofuel production [1]. Moreover, soybean is one of the most important commodities for exporter countries, and it is one of the most important food staples of importer countries. World production reached 312 million tons in the 2015/2016 season harvested from 119 million hectares [2]. Brazil is the second worldwide soybean producer behind the United States, with a production in the 2015/2016 season reaching 95 million tons harvested from 33 million hectares [3].

Although the worldwide soybean production is high, the yield is not suitable (2.62 tons ha^-1^). One of the most important reasons for the lack of yield is disease. Currently, more than 100 pathogens infect soybean plants. A disease that stands out, mainly in South America, is Asian soybean rust (SBR). This disease is caused by the fungal pathogen *Phakopsora pachyrhizi*. In Brazil, this disease has caused more than R$40 billion in crop losses since it was first detected in 2001 [4, 5]. Finding natural products that work in disease control has a number of economic and environmental benefits. The global threat to crop productivity represented by gap between agricultural production and food demand is steadily increasing year by year [6, 7]. Three strategies have been used to control SBR: applying chemical fungicides, employing specific cultivation practices, and most recently breeding or engineering of SBR-resistant soybean cultivars [8–10].

Several compounds may influence *P*. *pachyrhizi* infection. Isoflavonoids such as daidzein, genistein, and glyceollin efficiently reduce *P*. *pachyrhizi* uredospore germination in vitro [11]. Chung and Singh [12] described that SBR resistance in a *Glycine tomentella* accession was correlated with the presence of a flavonoid that also inhibited *P*. *pachyrhizi* spore germination. The high potential of phytoalexins in defeating SBR is further supported by medicarpin accumulating in *P*. *pachyrhizi* infected *Medicago truncatula*, a non-host of *P*. *pachyrhizi* [13]. SBR also reduces soybean photosynthetic function in susceptible soybean plants. Since resistant lines have reduced fungal growth, the resistance genes may protect these plants against injury to leaf photosynthesis [14]. Therefore, research evaluating phenolic compounds, proteins and compounds involved in photosynthesis is needed to understand the mechanism of *P*. *pachyrhizi* infection in soybean, and will consequently help breeders select resistant genotypes.

An important analysis is to identify how many measurements should be done to get a suitable accuracy to obtain useful results. This analysis is performed by using a repeatability coefficient [15]. The repeatability coefficient measures the ability of organisms to repeat character expression over a long period. This allows the verification of genotype behavior during the breeding cycle for different traits. High values of repeatability coefficients predict that the breeding value can be based on a few measurements [16].

The repeatability coefficient can be influenced by the genetic architecture of the trait and the environmental conditions under which the experiment is performed [15]; these factors make the study of repeatability coefficients important to estimate how many measurements should be done for each trait to guarantee a high accuracy. Several authors have investigated the changes related to enzymatic activities and photosynthetic compounds in soybean plants infected by *Phakopsora pachyrhizi* [17–20]. Although the biochemical and physiological changes caused by *P*. *pachyrhizi* in soybean plants are well documented, these studies have measured traits such as enzymatic activity, phenolic compound content and gas exchange at different hours after inoculation, that is, they use different measurements for these traits. To date, there are no known referenced studies that investigate how many measurements are needed to accurately assess enzymatic activities and photosynthetic compounds in soybean. Therefore, the objectives of this study were: (i) to determine the repeatability coefficients of the 14 traits evaluated in soybean plants inoculated with *P*. *pachyrhizi*; and (ii) to establish, based on the coefficient of repeatability and determination, the minimum number of measurements needed to predict the breeding value for each trait.

## Materials and methods

### Experimental design

The experiments were performed in the greenhouse of EMBRAPA Soybean, Londrina, State of Parana, Brazil. Climate conditions during the experiment are displayed in S1 Fig. The genotype BRS 361 was used in this study as a susceptible control to Asian Rust Soybean (SBR). Seeds were obtained from the germplasm bank in the EMBRAPA Soybean. Plants were maintained in the greenhouse through the V5 stage when different chemical products were applied using a CO_2_ pressurized sprayer.

Experiments were designed in a 3x2 factorial arrangement, with three treatments (water, Tween 20 and methyl jasmonate), and two inoculations (plants inoculated or non-inoculated). Each plant was evaluated four times (48, 96, 144, and 192 hours after inoculation—HAI). Experiments were performed in a randomized block design with six replications, and each plot was composed of one pot with five plants.

### Chemical products used

The chemical products used were: water as control treatment; a 0.02% surfactant (Tween 20—polyoxyethylene (20) sorbitan monolaurate; and 1.25 mM methyl jasmonate plus Tween 20 (Methyl jasmonate + tween 20). Methyl jasmonate is known as a regulator volatile [21], therefore plants treated with methyl jasmonate were maintained in a separate greenhouse for 24 hours using the same temperature and humidity to avoid affecting other treatments.

### Plant inoculation with *P*. *pachyrhizi*

Twenty-four hours after the application of the chemical products, soybean plants were inoculated. *P*. *pachyrhizi* inoculum was primarily maintained in soybean cultivar BRS 316 in the greenhouse of EMBRAPA soybean. Spores were collected with light beats on leaves with urediniospores onto white paper and then packed in microtubes. A suspension was then made with distilled water and 0.01% Tween 20 and adjusted to a final concentration of 1.4x10^5^ urediniospores/mL. After the inoculation, plants were maintained on nebulization to stimulate the infection, with the temperature ranging from 23°C to 25°C, humidity greater than 95% in the dark for 12 hours. Non-inoculated plants were treated with water + Tween 20 and maintained in the same conditions as inoculated plants.

### Evaluation of the SBR severity

The SBR severity was evaluated 16 days after inoculation in soybean plants in the V2 stage using the scale proposed by Godoy, Koga [22]. This scale is based on the percentage of infected leaf area, and ranged from 0.6% to 78.5% (S2 Fig).

### Enzymatic activity evaluation

Leaves from plants inoculated and non-inoculated with *P*. *Pachyrhizi* in the V4 and V5 stages were collected to determine enzymatic activities. Samples were composed of two leaves, and samples were collected at four times: 48, 96, 144, and 192 hours after inoculation (HAI). Samples were packed individually in aluminum foil packages, immediately frozen in liquid nitrogen and then stored in the ultrafreezer (-80°C). Chitinase (CHI) and β-1,3-glucanase (GLU) activities were determined. The total protein concentration for each sample was calculated following the Bradford method Bradford [23].

Foliar tissue was crushed in a pillowcase using liquid nitrogen plus 2% polyvinylpolypyrrolidone. The leaf extract was obtained by maceration of 0.3 g foliar tissue. The leaf powder was homogenized using 2 mL of 50 mM sodium phosphate buffer, pH = 6.5 and 1 mM phenylmethylsulfonyl fluoride. This mixture was centrifuged for 25 min at 20,000 X g at a temperature of 4°C. The supernatant was used to determine the enzymatic activity.

Chitinase activity was determined according to the method proposed by Roberts and Selitrennikoff [24] and modified by Harman, Hayes [25], in which p-nitrophenyl-β-D-N,N'-diacetylchitobiose (PNP) was used as a substrate. The incubation system consisted of the addition of a 20 μL aliquot of the supernatant obtained as described above, with 470 μL of 50 mM sodium acetate buffer, pH 5.0, and 10 μL of 2 mg mL^-1^ PNP. This mixture was stored at 37°C for 2 hours. The reaction was stopped by adding 0.5 mL of 0.2 M sodium carbonate and the absorbances were determined in the wave-length of 410 nm. A molar extinction coefficient of 7 x 104 mM^-1^.cm^-1^ was used to calculate the chitinase activity expressed in mM of the p-nitrophenyl produced per min^-1^ mg^-1^ protein.

β-1,3-glucanase activity was determined according to the method described by Lever [26], with a slight modification: 3,5-dinitrosalicylic acid substituted P-hydroxybenzoic acid [27]. Reaction was composed of 230 μL of 100 mM sodium acetate buffer, pH 5.0, 250 μL of 4 mg mL^-1^ laminarin substrate solution and 20 μL of foliar extract. This mixture was incubated for 30 min at 45°C, followed by adding 1 mL of 3,5-dinitrosalicylic acid, and heated to 100°C for 5 min. In the next step, the mixture was cooled to 30°C and the absorbance was determined by a wavelength of 540 nm. The results were expressed in units of absorbance.min^-1^.mg^-1^ per protein.

### Phenolic compound analysis

Samples for phenolic compound analysis were composed of V5 soybean leaves collected 48, 96, 144 and 192 HAI. Inoculated and non-inoculated plants were collected in the same period. Samples were stored individually in the aluminum foil packages in liquid nitrogen in the freezer (-20°C) through the extraction and analysis. Phenolic compounds were analyzed according to a standard methodology proposed by the laboratory of chemistry ecology of EMBRAPA Soybean. Foliar tissue was crushed in a pillowcase using liquid nitrogen and stored in a falcon tube. Five hundred mg of leaves were then weighed and mixed with 5 mL of 90% methanol and conditioned in an ultrasonic bath for 20 min. Samples were centrifuged for 12 min at 9,880 G at a temperature of 4°C, dried under vacuum, solubilized using 1.5 mL of 80% methanol and homogenized manually. After that, leaf extract was filtered using a Millipore® 0.45 μm membrane in a high performance liquid chromatography (HPLC), Shimadzu, Prominence model.

The methanol extracts from the samples were analyzed using a C18 column (250 mm long and 4.6 mm internal diameter, 5 μM particles). Aliquots of 10 μL were injected automatically; the instrument was equipped with a CBM-20ª controller, SPD-20A detector, DGU 20A5 degasser, LC-20AT pump, SIL-20A automatic sampler and CTO 20A furnace. The mobile phase was composed of two solvents: (A) 2% acetic acid (HOAc) and (B) A mixture of methanol, acetic acid and MilliQ® water (MeOH:HOAc:H_2_O; 18:1:1). The linear gradient system used in the analysis began with 75% of solvent A and 25% of solvent B, and by 40 min, the solvents were inverted using 25% of solvent A and 75% of solvent B for 5 min. After 45 min, it returned to the initial condition where it remained for 5 min before the next injection. The solvent flow was 1 mL min^-1^ and ultraviolet (UV) analysis was obtained at wavelengths of 260 and 280 nm.

Concentration of isoflavone aglycones (Genistein–GEI in μg.g^-1^) in the form 7-O-glycosides (daidzein–DZI in μg.g^-1^, and genistin–GI in μg.g^-1^), glycosidic malonyl (malonyl daidzin–MD in μg.g^-1^, and malonyl genistin–MG in μg.g^-1^), flavonoid (quercetin-3-O-rutinoside–R in μg.g^-1^) and phytoalexin coumestrol (C in μg.g^-1^) were identified based on spectra comparison between standards spectra, sample spectra, and retention time.

For each compound, the mean peak area was measured and multiplied by the correction factor based on each standard compound, which was the initial tissue weight and volume of solubilization. Different standard concentrations of isoflavones and phenolic acids (6,25; 12,5; 25; 50; 100 μg.mL^-1^) were tested in the HPLC to obtain the correction factor for each compound, which led to a dispersion graphic that obtained a linear regression equation and the correction factor.

### Gas exchange and chlorophyll pigment evaluation

Gas exchange and chlorophyll pigment evaluations were performed in the central V4 stage leaflets, previously at 39, 87, 135, and 183 HAI, between 9 am and 10 am. The leaf gas exchange parameters net CO_2_ assimilation rate (A), stomatal conductance to water vapor (GS), internal CO_2_ concentration (Ci) and transpiration rate (E) were measured by using a portable open-flow gas exchange system (LCpro-SD; ADA from Laboratory of Ecophysiology Vegetal in the EMBRAPA Soybean). At those periods, A was at its maximum under artificial photosynthetically active radiation (i.e., 1044 μmol photons m^-2^.s^-1^ at the leaf level).

Chlorophyll pigment contents were estimated indirectly by the SPAD index, calculated using a chlorophyll meter SPAD-502 and the following equation. The equation was generated based on calibration and validation tests performed by EMBRAPA soybean.

CLO=((SPAD*0,0007)−0,0071)

In which: CLO is the chlorophyll contents expressed in mg.cm^-2^.

### Statistical analysis

Analysis of variance was performed for each trait according to the following statistical model:

$$Y_{ij}=\mu +B_{j}+T_{i}+M_{k}+{TM}_{ik}+\varepsilon_{ij}$$

In which: Y_ij_ is the phenotypic value in the j^tth^ replication, evaluated in the i^th^ treatment; μ is the overall mean; B_j_ is the fixed effect of the j^th^ replication; T_i_ is the random effect of the i^th^ treatment; M_k_ is the random effect of the k^th^ measurement; TxM_ik_ is the interaction between the i^th^ treatment with the k^th^ measurement; and e_ij_ is the random error associated with the phenotypic value Y_ij_.

After that, five methods were used to estimate the repeatability coefficient: analysis of variance, principal component analysis based on the correlation matrix, principal component analysis based on the covariance matrix, structural analysis based on the correlation matrix, and mixed model.

Analysis of variance was calculated based on the following model:

$$y_{ij}=\mu +T_{i}+A_{j}+{TA}_{ij}+\varepsilon_{ij}$$

In which: *y*_*ij*_ is the phenotypic value for the i^th^ treatment in the j^th^ environment; *μ* é the general mean; *T*_*i*_ is the random effect of the i^th^ treatment influenced by permanent environment (i = 1, 2,…,p); *A*_*j*_ is the random effect of the temporal environment in the j^th^ measurement; *TA*_*ij*_ is the interaction between the i^th^ treatment influenced by permanent environment and the temporal environment in the j^th^ measurement; and *ε*_*ij*_ is the residual vector.

Heritability (h^2^) for each trait in each measurement was estimated by:

$$h^{2}=\frac{\sigma_{g}^{2}}{\sigma_{g}^{2}+\sigma_{e}^{2}}$$

In which: $\sigma_{g}^{2}$ is the genetic variance and $\sigma_{e}^{2}$ is the residual variance.

The repeatability coefficient (r) by analysis of variance was estimated by:

$$r=\frac{\sigma_{g}^{2}}{\sigma_{g}^{2}+\sigma_{e}^{2}}$$

The repeatability coefficient based on principal component analysis associated with correlation matrix was estimated by:

$$r=\frac{\lambda_{1}-1}{\eta -1}$$

In which: *λ*_1_ is the highest eigenvalue of the correlation matrix (R) associated with eigenvector in which all elements have the same direction and similar magnitude, and *η* is the number of measurements.

*λ*_1_ is estimated by:

$$\lambda_{1}=1+(\eta -1)\rho$$

in which: *ρ* is the correlation between two measurements based on the correlation matrix (R).

$$R=\left[\begin{array}{c} \begin{array}{ccc} 1 & \rho & \rho \\ \rho & 1 & \rho \\ \cdots & \cdots & \cdots \end{array}\\ \begin{array}{ccc} \rho & \rho & 1 \end{array} \end{array}\right]$$

In which: the R dimension is *η* x *η*.

The repeatability coefficient based on principal component analysis associated with covariance matrix is estimated by:

$$r=\frac{\lambda_{1}-{\hat{\sigma }}_{y}^{2}}{{\hat{\sigma }}_{y}^{2}(\eta -1)}$$

In which: ${\hat{\sigma }}_{y}^{2}$ is the phenotypic variance.

In this case, *λ*_1_ is the highest eigenvalue of the covariance matrix (Γ) associated with eigenvector which all elements have the same direction and similar magnitude.

$$\Gamma ={\hat{\sigma }}_{y}^{2}\left[\begin{array}{c} \begin{array}{ccc} 1 & \rho & \rho \\ \rho & 1 & \rho \\ \cdots & \cdots & \cdots \end{array}\\ \begin{array}{ccc} \rho & \rho & 1 \end{array} \end{array}\right]$$

and *λ*_1_ is estimated by:

$$\lambda_{1}={\hat{\sigma }}_{y}^{2}[1+(\eta -1)\rho ]$$

The repeatability coefficient based on structural analysis associated with the correlation matrix is estimated by:

$$r=\frac{2}{\eta (\eta -1)}\sum_{j<}\sum_{j\prime }r_{jj^{\prime }}$$

In which *r*_*jj*′_ is the phenotypic correlation between treatments during the measurements.

Mixed model equations were used according to Resende [28] using the software Selegen [29]

Estimation of the number of measurements needed to obtain a certain coefficient of determination, the following equation was used:

$$R^{2}=\frac{\eta r}{1+r(\eta -1)}$$

Analyses were performed using software R [30] and GENES [31].

## Results

### Analysis of variance and estimation of genetic and environmental parameters

The analysis of variance was performed to verify significant differences among treatments for 14 traits evaluated in soybean plants inoculated or non-inoculated with *Phakopsora pachyrhizi* during four consecutive measurements (48, 96, 144, 192 hours after inoculation—HAI). Significant differences were observed for all traits except Ci for the treatment effect (Table 1). The measurement effects of all traits were significantly different. In addition, significant differences were found for all traits for the interaction Treatment x Measurement, except C, CHI and CLO.

**Table 1:** Analysis of variance for the 14 traits evaluated in the soybean plants inoculated or non-inoculated with *Phakopsora pachyrhizi* during four consecutive measurements (48, 96, 144, 192 hours after inoculation).

Trait	MSTrat	MSMea	MSTratxMea	MSRes
**DZI**	6175.67**	849.71**	2762.43**	110.78
**GI**	2312.22**	2590.58**	826.39**	224.19
**MD**	110494.54**	16210.89**	41313.28**	607.88
**R**	2077.38**	742.43**	284.12*	137.87
**MG**	49942.03**	27327.93**	20220.51**	551.27
**GEI**	1.39**	3.56**	1.34**	0.12
**C**	18.98**	3.03**	3.30^ns^	0.50
**GLU**	33997.61**	5503.93**	776.32*	380.52
**CHI**	754.57**	349.31**	6.66^ns^	6.44
**CLO**	4x10^-5^*	2.1x10^-4^**	2x10^-5^^ns^	2x10^-5^
**Ci**	6574.69^ns^	94792.24**	6555.60*	3620.35
**E**	1.26**	3.19**	0.46**	0.11
**GS**	0.46**	0.10*	0.13**	0.03
**A**	67.07**	247.01**	3.79*	3.49

MSTrat–Mean Squares for treatment; MSMea–Mean Squares for measurement; MSTratxMea–Mean squares for the interaction TreatmentxMeasurement; DZI—Daidzein; GI—Gensitin; MD–Malonyl Daidzin; R—Quercetin-3-O-Rutinoside; MG–Malonyl Genistin; GEI–Genistein; C–Coumestrol; GLU– β-1,3-glucanase; CHI—Chitinase; CLO–Chlorophyll; Ci—internal CO_2_ concentration; E—transpiration rate; GS—stomatal conductance to water vapor; and A—CO_2_ assimilation rate

^ns^: not significance

*: significant at 5% probability

**: significant at 1% probability.

It was observed that the mean decreased with the plant growth for GI, GLU, and CHI depending on the different measurements (Table 2). In addition, the mean for DZI, MD, MG, C, and Ci decreased as well, except for the second measurement when the mean was lower than the third measurement. The mean for all traits decreased when we compared the first and the fourth measurements, except for CLO which was constant during the measurements.

**Table 2:** Mean, heritability (h^2^) and coefficient of variation (CV, %) for the 14 traits evaluated in soybean plants inoculated or non-inoculated with *Phakopsora pachyrhizi* during four consecutive measurements (48, 96, 144, 192 hours after inoculation).

Trait	Mean				h^2^				CV (%)			
	48	96	144	192	48	96	144	192	48	96	144	192
**DZI**	22.04	11.35	18.76	13.58	0.98	0.94	0.95	0.94	47.26	103.57	67.47	61.93
**GI**	70.06	59.05	58.44	49.34	0.93	0.82	0.71	0.63	15.56	24.38	22.13	39.19
**MD**	97.36	49.59	86.41	66.33	0.99	0.88	0.99	0.98	30.06	47.95	28.01	37.72
**R**	34.68	23.78	27.36	28.18	0.88	0.53	0.95	0.58	31.34	56.27	26.46	45.05
**MG**	125.92	77.22	84.66	61.27	0.98	0.96	0.96	0.95	22.83	25.54	28.93	35.73
**GEI**	0.60	1.27	0.97	.049	0.86	0.73	0.96	0.80	45.80	36.59	36.61	70.11
**C**	1.22	1.28	0.96	0.64	0.96	0.95	0.57	0.82	60.01	53.56	81.76	95.63
**GLU**	132.57	123.07	122.65	103.12	0.95	0.96	0.95	0.96	17.09	15.89	11.98	17.12
**CHI**	20.27	18.95	18.35	13.17	0.97	0.97	0.96	0.96	12.17	12.83	14.13	17.43
**CLO**	0.01	0.01	0.01	0.01	0.24	0.11	0.20	.053	30.94	10.00	35.77	32.85
**Ci**	419.16	296.05	376.61	374.30	0.63	0.79	0.41	0.81	3.67	6.48	31.75	3.23
**E**	2.21	2.25	1.69	1.74	0.49	0.97	0.47	0.99	6.17	6.88	33.75	5.15
**GS**	0.58	0.52	0.62	0.50	0.75	0.86	0.83	0.96	19.15	27.65	44.77	15.94
**A**	13.77	14.90	8.97	11.53	0.11	0.84	0.80	0.93	15.67	13.05	21.65	11.56

DZI—Daidzein; GI—Gensitin; MD–Malonyl Daidzin; R—Quercetin-3-O-Rutinoside; MG–Malonyl Genistin; GEI–Genistein; C–Coumestrol; GLU– β-1,3-glucanase; CHI—Chitinase; CLO–Chlorophyll; Ci—internal CO_2_ concentration; E—transpiration rate; GS—stomatal conductance to water vapor; and A—CO_2_ assimilation rate.

Heritability was high (greater than 0.70) for all traits except CLO in all measurements: R in the second measurement, C in the third measurement, Ci and E in the first and third measurements, and A in the first measurement (Table 2). The highest heritability was observed for GLU and CHI, which were greater than 0.95 for all measurements.

The coefficients of variation were low (CV less than 20%) for GLU and CHI for all measurements, and Ci, E and A for the first, second and fourth measurements (Table 2). In addition, the coefficients of variation were medium (ranged from 21% from 40%) in all measurements for the GI, MG, CLO. However, at least one measurement had a coefficient of variation greater than 41% for other traits.

### Estimate of repeatability coefficients and coefficients of determination

Five methods were used to estimate the repeatability coefficients and coefficients of determination for 14 traits evaluated in soybean plants inoculated or non-inoculated with *P*. *pachyrhizi* during four consecutive measurements (48, 96, 144, 192 hours after inoculation). The principal component analysis based on the covariance matrix out-performed other methods for all traits, except MD in which the principal component analysis based on the correlation matrix showed the highest repeatability coefficient (Table 3).

**Table 3:** Repeatability coefficients (r) and coefficients of determination (R^2^) obtained by different statistical methods for 14 traits evaluated in soybean plants inoculated or non-inoculated with *Phakopsora pachyrhizi* during four consecutive measurements (48, 96, 144, 192 hours after inoculation).

Trait	ANOVA		PCA-COV		PCA-COR		Structural		MM	
	r	R^2^	r	R^2^	r	R^2^	r	R^2^	r	R^2^
**DZI**	0.24	56.85	0.54	82.67	0.34	68.07	0.33	66.70	0.35	0.68
**GI**	0.37	70.86	0.57	84.13	0.42	74.37	0.33	67.08	0.22	0.52
**MD**	0.30	63.66	0.42	74.57	0.46	77.73	0.45	77.17	0.45	0.76
**R**	0.61	86.69	0.70	90.53	0.67	89.06	0.66	88.76	0.37	0.70
**MG**	0.28	60.87	0.62	87.05	0.37	70.76	0.35	68.81	0.38	0.71
**GEI**	0.01	2.75	0.49	79.54	0.25	58.18	0.02	10.90	0.16	0.43
**C**	0.50	80.54	0.87	96.53	0.56	84.05	0.54	82.60	0.46	0.77
**GLU**	0.88	96.91	0.92	98.05	0.92	98.12	0.92	98.10	0.77	0.93
**CHI**	0.95	98.91	0.96	99.05	0.96	99.07	0.96	99.07	0.84	0.95
**CLO**	0.68	89.72	0.72	91.26	0.72	91.30	0.72	91.26	0.32	0.65
**Ci**	0.01	0.69	0.89	97.25	0.41	74.24	0.16	70.90	0.04	0.14
**E**	0.37	70.28	0.86	96.11	0.77	93.24	0.01	4.58	0.27	0.60
**GS**	0.44	76.02	0.78	93.65	0.55	83.22	0.35	68.78	0.29	0.62
**A**	0.71	91.12	0.87	96.43	0.73	91.85	0.77	91.22	0.45	0.77

DZI—Daidzein; GI—Gensitin; MD–Malonyl Daidzin; R—Quercetin-3-O-Rutinoside; MG–Malonyl Genistin; GEI–Genistein; C–Coumestrol; GLU– β-1,3-glucanase; CHI—Chitinase; CLO–Chlorophyll; Ci—internal CO_2_ concentration; E—transpiration rate; GS—stomatal conductance to water vapor; and A—CO_2_ assimilation rate.

On the other hand, the repeatability coefficient estimated by mixed model and ANOVA were the lowest compared to principal component analysis and structural analysis. The same shape was observed for the coefficient of determination, which principal component analysis based on the covariance matrix estimated the highest values for almost all traits, followed by principal component analysis based on the correlation matrix and structural analysis (Table 3).

### Calculating the number of measurements needed to obtain a reasonable coefficient of determination

An estimate of the optimal number of measurements needed to obtain an acceptable coefficient of determination was calculated from one to ten measurements for each trait and plotted.

**Fig 1:**
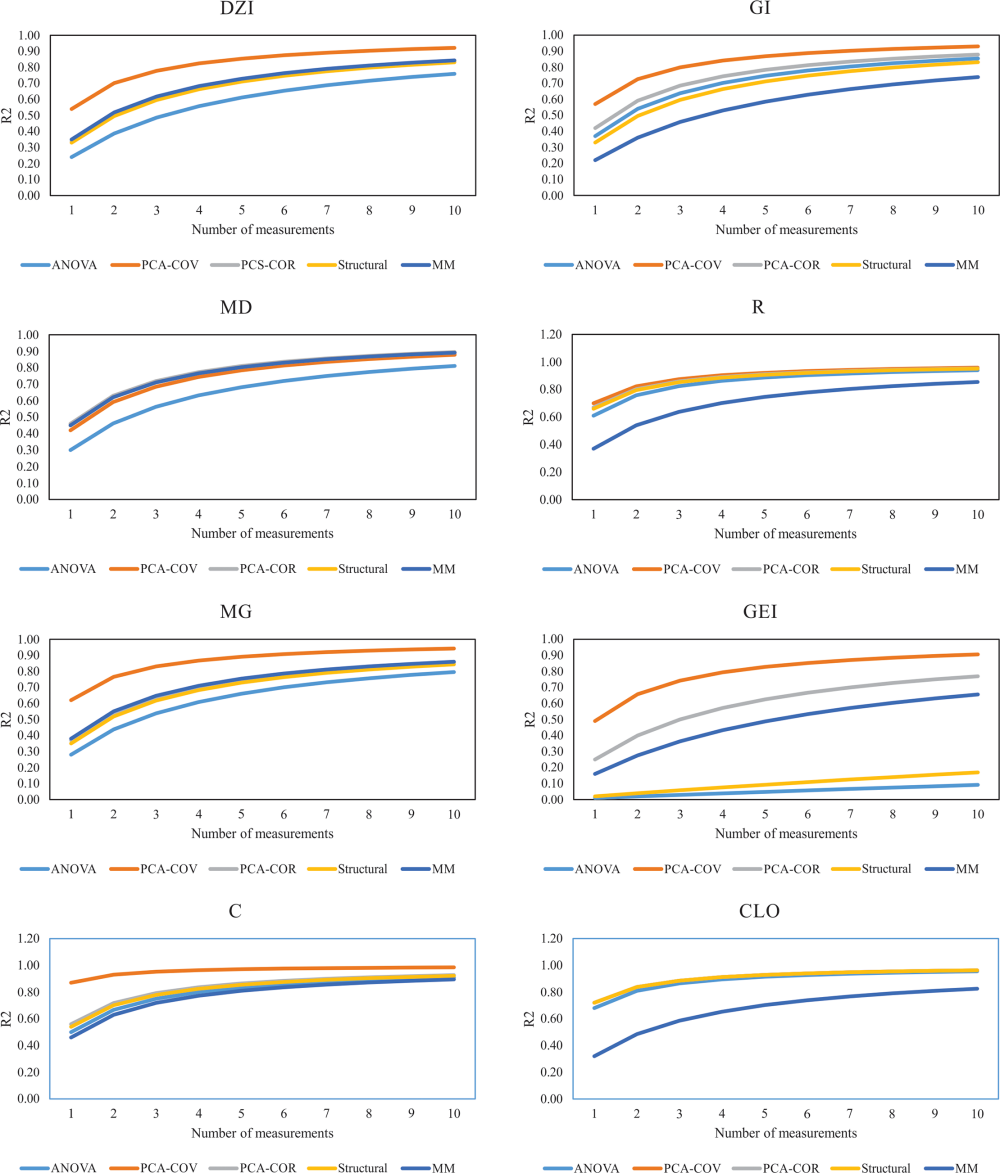
Graphic analysis of the minimum number of measurements required to reach a determined degree of certainty for phenolic compounds and chlorophyll according to different methods: ANOVA–analysis of variance; PCA-COV—principal component analysis associated with covariance matrix; PCA-COR—principal component analysis associated with correlation matrix; structural analysis; MM–mixed model. DZI—Daidzein; GI—Gensitin; MD–Malonyl Daidzin; R—Quercetin-3-O-Rutinoside; MG–Malonyl Genistin; GEI–Genistein; C–Coumestrol; and CLO–Chlorophyll.

**Fig 2:**
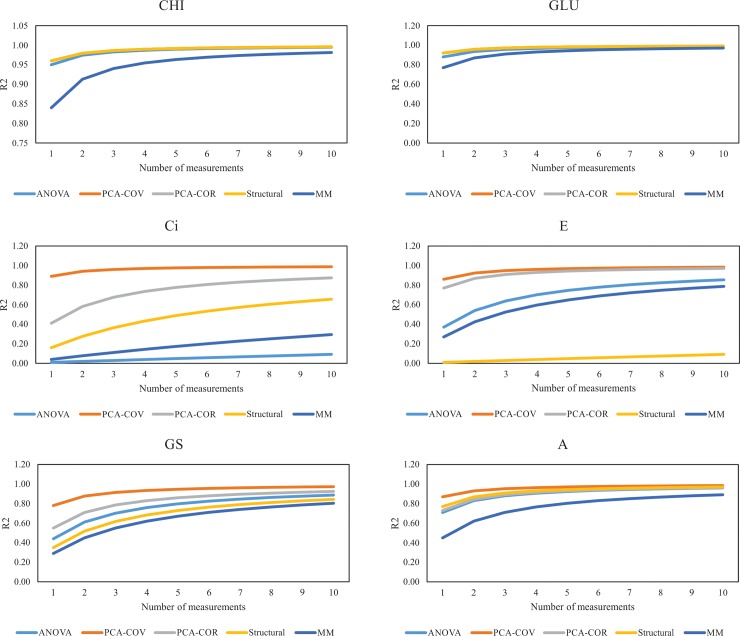
Graphic analysis of the minimum number of measurements required to reach a determined degree of certainty for enzymatic activities and photosynthetic performance according to different methods: ANOVA–analysis of variance; PCA-COV—principal component analysis associated with covariance matrix; PCA-COR—principal component analysis associated with correlation matrix; structural analysis; MM–mixed model. GLU– β-1,3-glucanase; CHI—Chitinase; Ci—internal CO_2_ concentration; E—transpiration rate; GS—stomatal conductance to water vapor; and A—CO_2_ assimilation rate.

The coefficients of determination were different among methods for the same number of measurements (Figs 1 and 2). Four measurements were suitable to obtain a coefficient of determination higher than 0.7 for almost all traits (except MD) based on principal component analysis via covariance matrix. Moreover, six measurements were enough to obtain a coefficient of determination greater than 0.7 based on principal component analysis via correlation matrix for all traits (Figs 1 and 2).

Based on ANOVA and mixed models, more than 10 measurements were needed to obtain a coefficient of determination greater than 0.70 for DZI, Gi, GEI, CLO, Ci, E and GS (Figs 1 and 2). GLU and CHI were the traits that needed fewer measurements (just one) to obtain a coefficient of determination greater than 0.70, followed by C and A with four and five measurements respectively.

## Discussion

### Analysis of variance and estimation of genetic and environmental parameters

There were significant treatment effects for all traits, except Ci (Table 1). This indicates that the inoculation of *P*. *pachyrhizi* made changes to the enzymatic activities, phenolic compounds, and gas exchange in soybean plants. Therefore, it is important to understand the biochemical and physiological mechanisms and their relation to the Soybeanx ASR interaction because it will help breeders to select resistant genotypes or to find methodologies that will help the farmers to manage the pathogen more efficiently.

Findings in this study agree with previous research which proved that methyl jasmonate can efficiently reduce disease severity in many crops such as sweet cherry fruit [32], *Alternaria brassicicola*, *Botrytis cinerea* and *Plectosphaerella cucumerina* in *Arabidopsis thaliana* [33], *Monilinia fructicola* in peach [34], *Erysiphe necator* in grapes [35] and *Colletotrichum acutatum* in plum [36]. Moreover, methyl jasmonate can affect disease severity in two ways: directly via fungal growth suppression as well as spore germination suppression, which were reported for *Alternaria alternata* [37], and indirectly via fungal growth suppression as observed in *A*. *brassicicola* on *Arabidopsis thaliana* [38].

The significant effects of the measurement factors for all traits indicated that the concentration of those substances changed over time. These results were expected because the fungal latency period likely increased several physiological changes due to the high spore production that consequently caused changes in the values of the traits (Table 2). These results reinforce the need to study how many measurements are suitable to select superior genotypes in ASR-tolerant soybean, high yield, and other important agronomic traits. In addition, the evaluation of enzymatic activities, phenolic compounds and gas exchange is necessary to better understand how ASR can affect yield and possibly use these traits to indirectly select ASR-resistant genotypes.

The estimation of heritability allows breeders to know the fraction of phenotypic variation among treatments is explained by genetic factors [15]. The estimation of this parameter presented high or moderate magnitude for all traits, except CLO (Table 2). These results indicated that the CLO evaluation can be significantly influenced by environmental conditions.

The measurement 48 hours after inoculation performed the smallest CV estimate (Table 2). However, evaluating biochemical and physiological traits in advanced infected stages can contribute most to the discrimination of resistant genotypes for *P*. *pachyrhizi*, although the experimental precision decreased during the measurements. Repeatability analysis can be used to fix the problem of experimental precision since repeatability coefficients and coefficients of determination estimated by several methodologies can indicate how many measurements are needed to obtain a suitable reliability for each trait [16].

### Estimate of repeatability coefficients and coefficients of determination

Principal component analysis based on the covariance matrix estimated the highest repeatability coefficient for all traits, except MD; this resulted in fewer measurements needed to identify superior soybean genotypes for traits related with ASR infection. These results are similar to the results observed by Matsuo, Sediyama [39] that evaluated the repeatability of oidium (*Erysiphe diffusa*) severity in several soybean genotypes. In addition, several studies revealed the efficiency of the principal component analysis based on the covariance matrix to estimate the repeatability coefficients for agronomic traits in perennial species [40–43] and annual species [44–46]. This type of analysis can help breeders, physiologists and phytopathologists to find experimental strategies to evaluate traits with accurate results.

The significance of the measurement effect indicates that many changes occurred in the physiological and biochemical mechanisms of the soybean plants during the evaluation [15]. This effect varied among treatments, and ANOVA did not allow the separate analysis of this effect; therefore, it was consequently mixed with the residual. This fact can lead to the underestimation of the repeatability and consequently the overestimation of the number of measurements needed. Therefore, principal component analysis that considers cyclic trait behavior is most recommended for the estimation of the repeatability coefficient. This is because the eigenvector estimated by principal component analysis expresses the progeny trend and their capacity to maintain their relative position during the evaluation period [47].

This type of analysis might be very important to understand how the physiological changes occur in soybean plants affected by ASR over a period of time. For instance, according to Cruz, Rodrigues [48] chitinase and β‑1,3‑glucanase are important to decrease the *P*. *pachyrhizi* colonization in soybean leaf tissue. These enzymes are involved in plant defense against the pathogen by the chitin and β-1,3-glucan hydrolytic action present on the fungal cell wall, and consequently the oligosaccharide liberation that may act as elicitors of the plant defense mechanism against the pathogen [49]. The overexpression of genes affecting chitinase and β‑1,3‑glucanase in plants have increased the resistance to several plant pathogens [50].

### Number of measurements needed to obtain a reasonable coefficient of determination

Sustainable food production to feed a growing world population is a major challenge for plant scientists, especially given the unpredictable and dynamic nature of global climatic conditions. These changes affect the dynamics of diseases in crops such as soybeans. Therefore, the development of effective strategies to assess the severity of these diseases will be essential for their control in a sustainable way [6, 7].

A coefficient of determination greater than 0.70 is desirable in plant breeding because it improves the selection accuracy, i.e., breeders can select superior genotypes more accurately. Based on this parameter, the method of principal component analysis based on the covariance matrix indicated that six measurements are needed for all traits. These results are similar to results reported by Matsuo, Sediyama [39], which concluded that four measurements are needed to obtain a suitable prediction, i.e., a prediction with a confidence greater than 70% for the severity in the foliates infected by oidium.

Based on the results revealed in this study, breeders, physiologists and phytopathologists will be able to evaluate phenolic compounds, enzymatic activities and gas exchange more accurately to select superior soybean genotypes for resistance to *P*. *pachyrhizi*. Additional research should be done evaluating these traits in several genotypes and the selection of resistant genotypes based on these traits.

## Conclusion

The number of measurements affect the reliability in traits related to enzymatic activity, phenolic compound content and gas exchange in soybean plants infected by Asian rust soybean. Six measurements are suitable to obtain a coefficient of determination higher than 0.7 for all traits based on principal component analysis.

## Supporting information

S1 Fig Daily variation of air temperature and solar radiation in June 2013. The days in which gas exchange was evaluated were highlighted.
(TIF)

S2 Fig Diagrammatic scale of the Asian rust soybean severity by Godoy, Koga [22]. (TIF)
