# Correction: The number of measurements needed to obtain high reliability for traits related to enzymatic activities and photosynthetic compounds in soybean plants infected with *Phakopsora pachyrhizi*

**DOI:** 10.1371/journal.pone.0206485

**Published:** 2018-10-24

**Authors:** Tássia Boeno de Oliveira, Leonardo de Azevedo Peixoto, Paulo Eduardo Teodoro, Amauri Alves de Alvarenga, Leonardo Lopes Bhering, Clara Beatriz Hoffmann-Campo

The sixth author’s name is spelled incorrectly. The correct name is: Clara Beatriz Hoffmann-Campo. The correct citation is: Oliveira TBd, Azevedo Peixoto Ld, Teodoro PE, Alvarenga AAd, Bhering LL, Hoffmann-Campo CB (2018) The number of measurements needed to obtain high reliability for traits related to enzymatic activities and photosynthetic compounds in soybean plants infected with *Phakopsora pachyrhizi*. PLoS ONE 13(2): e0192189. https://doi.org/10.1371/journal.pone.0192189
