# Supplementary figures and images for: The number of measurements needed to obtain high reliability for traits related to enzymatic activities and photosynthetic compounds in soybean plants infected with Phakopsora pachyrhizi
Source: PLoS One. 2018 Feb 13;13(2):e0192189. doi: 10.1371/journal.pone.0192189 (PMC5811010; doi:10.1371/journal.pone.0192189)

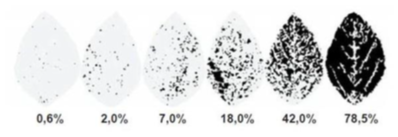

Supplement: S2 Fig — (TIF) [file pone.0192189.s002.tif]
